# Characteristics and diversity of mutations in regulatory genes of resistance-nodulation-cell division efflux pumps in association with drug-resistant clinical isolates of *Acinetobacter baumannii*

**DOI:** 10.1186/s13756-021-00924-9

**Published:** 2021-03-10

**Authors:** Bahare Salehi, Zohreh Ghalavand, Abbas Yadegar, Gita Eslami

**Affiliations:** 1 Infectious Diseases and Tropical Medicine Research Center, Shahid Beheshti University of Medical Sciences, Tehran, Iran; 2 Department of Microbiology, School of Medicine, Shahid Beheshti University of Medical Sciences, Tehran, Iran; 3 Foodborne and Waterborne Diseases Research Center, Research Institute for Gastroenterology and Liver Diseases, Shahid Beheshti University of Medical Sciences, Tehran, Iran

**Keywords:** *Acinetobacter baumannii*, RND-type efflux pumps, Efflux pumps inhibitor, Two-component systems

## Abstract

**Background:**

This study was aimed to characterize the genetic diversity and expression of three putative resistance-nodulation-cell division (RND)-type efflux systems and their contribution to multidrug efflux in clinical isolates of *Acinetobacter baumannii*.

**Methods:**

Antimicrobial susceptibility testing of 95 *A. baumannii* isolates was determined by Kirby-Bauer disk diffusion for 18 antibiotics and minimum inhibitory concentration (MIC) of colistin was determined by the broth microdilution method. Moreover, the MIC of five classes of antibiotics was assessed using E-test strips in the presence and absence of phenylalanine-arginine beta-naphthylamide (PAβN). Regulatory genes of the RND efflux pumps (*adeRS*, *adeL*, *adeN* and *baeSR*) were subjected to sequencing. The relative expression of *adeB*, *adeG* and *adeJ* genes was determined by quantitative real-time PCR (qRT-PCR).

**Results:**

Overall, the majority of isolates (94%) were extensively drug-resistant (XDR). In the phenotypic assay, efflux pump activity was observed in 40% of the isolates against multiple antibiotics mainly tigecycline. However, we found no efflux activity against imipenem. Several amino acid substitutions were detected in the products of regulatory genes; except in AdeN. Of note, G186V mutation in AdeS was found to be associated with overexpression of its efflux pump. No insertion sequences were detected.

**Conclusions:**

Our findings outlined the role of RND efflux pumps in resistance of *A. baumannii* to multiple antibiotics particularly tigecycline, and pointed out the importance of a variety of single mutations in the corresponding regulatory systems. Further studies are required to decipher the precise role of RND efflux pumps in multidrug-resistant clinical isolates of *A. baumannii*.

## Background

*Acinetobacter baumannii* has proved to be one of the highly resilient and adaptable superbugs that have the propensity to cause imminent nosocomial outbreaks worldwide [1]. The isolation of extensively drug-resistant *A. baumannii* (XDR-AB) has drastically limited and nullified most of the therapeutic armamentarium in healthcare settings [1–3]. Genomic plasticity and genetic variabilities; including mutations in endogenous structural or regulatory genes and insertion of mobile genetic elements, can account for the development of various resistance mechanisms in XDR-AB isolates. Besides, intrinsic resistance determinants such as chromosomally encoded carbapenemase genes, decreased membrane permeability, and efflux systems are inherent in *A. baumannii*. Of note, in clinical practice, alterations in efflux system regulators, in a single step, can boost efflux activity through overexpression and lead to the extrusion of a wide range of substrates [4–6]. Antibiotic extrusion occurs mainly through overexpression of efflux pumps. In particular, resistance-nodulation-cell division (RND) superfamily pumps are ubiquitous in Gram-negative bacteria and are remarkable for their ability to be selected after exposure to an antibiotic [7].

Overexpression of three chromosomally encoded *Acinetobacter* drug efflux (Ade) RND systems; AdeABC [8], AdeFGH [9], AdeIJK [10], has been reported in evolving multidrug-resistant (MDR) *A. baumannii* isolates [6, 11]. These tripartite assemblies of RND efflux pumps span across two membranes, and consist of an inner membrane located RND transport protein with a substrate-binding site that pumps drugs out utilizing proton motive force [8–10]. AdeRS two-component regulatory system (TCS) controls AdeABC expression, the LysR-type transcriptional regulator AdeL regulates AdeFGH, and the TetR transcriptional regulator AdeN represses AdeIJK. Point mutations, deletions, or insertions in each of these regulatory systems can affect their respective pump expression [9, 12–16].

Although several studies have showed the role of AdeRS in controlling the AdeABC efflux pump, some contradictory results have revealed no correlation between the overexpression of AdeABC pump and AdeRS mutations, elucidating that other mechanisms may be involved [14, 15, 17, 18]. BaeSR is another TCS that has been suggested to regulate AdeABC and likewise AdeIJK [19, 20]. TCSs are signal transduction pathways comprised of a sensor kinase (SK) and its cognate response regulator (RR), which are capable of sensing and mediating drastic and immediate adaptive responses to extracellular and/or intracellular stimuli leading to modulation of gene expression [21, 22].

The objective of the current study was to determine the genetic mutations in the cognate regulators of three Ade efflux systems, and their gene expression in XDR and MDR isolates of *A. baumannii* recovered from different clinical specimens at a Hospital in Tehran, Iran.

## Methods

### Bacterial isolates and identification

A total of 95 non-repetitive *A. baumannii* isolates were collected from inpatients mostly with respiratory tract infections, bacteraemia, and wound infections between August 2016 and February 2017 from an educational hospital in Tehran, Iran. Species identification was performed by the API20NE system (Biomérieux, Marcy-l’Étoile, France), and PCR amplification of the *bla*_OXA-51-like_ gene [23] and *rpoB* sequencing [24]. This study was approved by the local ethics committee of Shahid Beheshti University of Medical Sciences (IR.SBMU.MSP.REC.1396.22).

### Antimicrobial susceptibility testing (AST) and efflux pump activity determination

Antibiotic susceptibility profile was determined by Kirby-Bauer disk diffusion for 18 antibiotics (Mast Co., Merseyside, UK) on Mueller–Hinton (MH) agar (Merck, Germany), and minimum inhibitory concentration (MIC) of colistin was determined by broth microdilution method in Mueller–Hinton broth (Merck, Germany), as described by the clinical and laboratory standards institute (CLSI) [25]. Colistin sulfate powder was purchased from Sigma-Aldrich (St. Louis, MO, USA). *Escherichia coli* ATCC 25922 and *Pseudomonas aeruginosa* ATCC 27853 strains were used as quality control. Results were interpreted according to the CLSI guidelines [26].

For verification of RND efflux pumps activity, the MIC of agents from five classes of antibiotics including imipenem, levofloxacin, cefepime, tigecycline, and gentamicin were assessed with E-test strips with and without efflux pump inhibitor (EPI) phenylalanine-arginine beta-naphthylamide (PAβN) (Sigma, St. Louis, Mo., USA). PAβN was added to MH agar cooled to 50 °C at a final concentration of 25 µg/ml. Bacterial suspensions equal to a 0.5 McFarland standard were inoculated on MH agar and E-test strips were overlaid, then plates were incubated for 18 h at 37 °C. Four-fold or greater reduction of MIC in the presence of EPI was considered significant [27]. Due to lack of tigecycline breakpoints in CLSI for *A. baumannii*, the US FDA tigecycline susceptibility breakpoints for *Enterobacteriaceae* (susceptible ≤ 2 µg/ml; intermediate > 2 and < 8 µg/ml; resistant ≥ 8 µg/ml) were used as MIC interpretation criteria. *A. baumannii* isolates with non-susceptibility to ≥ 1 agent in ≥ 3 antimicrobial categories are considered as MDR, and isolates with non-susceptibility to ≥ 1 agent in all but ≤ 2 antimicrobial categories are defined as XDR [28].

### Primer design and PCR-sequencing of RND-type efflux pump regulatory genes

Screening of RND efflux transporters *adeB*, *adeG* and *adeJ*, and their regulatory genes *adeRS*, *adeL*, *adeN* and *baeSR* was examined by PCR. Briefly, isolates were propagated overnight in Luria–Bertani (LB) broth (Merck, Germany) at 37 °C to recover fresh cultures. Genomic DNA was extracted using the High Pure PCR Template Preparation Kit (Roche Co., Germany) according to the manufacturer’s instructions. Gene Runner software version 6.0.28 and Primer3 were used to design specific primers for amplification of full-length sequence for each regulatory gene. The primer sets were purchased from Bioneer Co. (Bioneer, Daejeon, South Korea) (Table 1). DNA sequencing was performed by the conventional Sanger sequencing method using the ABI 3730XL DNA Analyzer (Bioneer, Daejeon, South Korea). All sequences were analyzed using the NCBI BLAST program (https://blast.ncbi.nlm.nih.gov/Blast.cgi) and multiple-sequence alignment analyses were performed by using ClustalW2 at the European Bioinformatics Institute website (https://www.ebi.ac.uk/Tools/msa/clustalw2/) and BioEdit software version 7.2.5 [29].

**Table 1:** Primers used in this study

Target genes	Primer sequence (5′–3′)	Amplicon (bp)	References
PCR and sequencing			
*OXA-51*	F: TAATGCTTTGATCGGCCTTG R: TGGATTGCACTTCATCTTGG	353	[23]
*rpoB*	F: GTGATAARATGGCBGGTCGT R: CGBGCRTGCATYTTGTCRT	543	[24]
*adeB*	F: TTAACGATAGCGTTGTAACC R: TGAGCAGACAATGGAATAGT	541	[30]
*adeG*	F: TTCATCTAGCCAAGCAGAAG R: ATGTGGGCTAGCTAACGGC	652	[9]
*adeJ*	F: ATTGCACCACCAACCGTAAC R: TAGCTGGATCAAGCCAGATA	463	[30]
*adeR*	F: ATGAGTGTGTAGGGATAATC R: TACTACAGAAAATAGCGTAAC	961	This study
*adeS*^a^	F: ATGTTAATTAATGTGCGTGGC R: CAGCTTATATGTTAGGTGTCC	1315	This study
*adeS*	F: ATGAAAAGTAAGTTAGGAATTAGTAAG R: TTAGTTATTCATAGAAATTTTTATG	1074	[15]
*baeS1*^b^	F1: TAAAGATGAAGAAACAATGGAG R1: ATCACCTAACTCATCATTTCG	1024	This study
*baeS2*^b^	F2: CCAGTTTTAGATAAAGAAGATGC R2: ACCAGATGCGCTAACTCGAC	1014	This study
*baeR*	F: CATTAGGTGGTTTACGTTGTG R: CTGATGTCGCGGTGAGCTG	845	This study
*adeL*	F: TGGACGGAGCATAAAAGTTTG R: CAATTTCTATACCATAAGTTAAGG	1246	This study
*adeN*	F: AAACATACCAATGACCATCG R: GTAGCTACTCCATAATAATTG	842	This study
RT-PCR analysis			
*adeB*	F: AACGGACGACCATCTTTGAGTATT R: CAGTTGTTCCATTTCACGCATT	84	[15]
*adeG*	F: AACTATGCGGTGCTCAAC R: GGTGAATTACTTGGTGATGC	218	[5]
*adeJ*	F: AGCTGGTGCTATGGGCGTTA R: GCCACCCCATGCAATACG	64	[31]
*rpoB*	F: GAGTCTAATGGCGGTGGTTC R: ATTGCTTCATCTGCTGGTTG	110	[14]

^a^Primer sets designed in this study for *adeS* gene, but could not successfully amplify it

^b^Since the complete sequence of the *baeS* gene is 1659 bp, we designed two overlapping primer pairs for full-length amplification and sequencing of this gene

### Total RNA isolation and quantitative real-time PCR (qRT-PCR)

The expression of *adeB*, *adeG* and *adeJ* RND transporter genes was assessed by quantitative real-time RT-PCR. For preparation of total RNA, overnight bacterial cultures were subcultured 1:100 in fresh LB broth at 37˚C to mid-log phase (optical density at 600 nm [OD600]: ca*.* 0.6–0.8). After centrifugation at 8000 g for 5 min, bacterial pellet was resuspended in RNAlater (Sigma-Aldrich Ltd.), vortexed for 20 s and incubated for 15 min at room temperature for RNA stabilization. Total RNA was extracted using High Pure RNA isolation kit (Roche, Germany) as the manufacturer’s instructions. Extracted RNA samples were treated with DNase I (CinnaGen Co., Iran) to remove any residual DNA contaminations, and frozen at − 80 °C until used for expression analysis. The concentration and quality of RNA samples were assessed by measuring absorbance at 260 nm using a spectrophotometer (Denovix, DS-11, USA). Reverse transcription was carried out in a Mastercycler (Eppendorf, Hamburg, Germany) by using TaKaRa PrimeScript TM RT reagent kits (Takara Bio Inc., Kusatsu, Japan) according to the manufacturer’s protocol. Negative-control reactions with an equal amount of RNA sample and all the reagents but without reverse transcriptase were included. PCR amplifications were performed in a Rotor-Gene^®^ Q (Qiagen, Germany) real-time PCR system using BioFACT™ 2X Real-Time PCR Master Mix (BIOFACT, South Korea). Specific primers used for qRT-PCR are presented in Table 1. The PCR program was as follows: 95 °C for 15 min, followed by 40 cycles at 95˚C for 20 s, 56 °C for 30 s, and 72 °C for 20 s. A melting curve was run at the end of each qPCR run to ensure that there was only one peak and only one product for each primer pair. Relative expression was calculated by the 2^−ΔΔCT^ method and the RNA input was normalized against the housekeeping gene *rpoB*. The expression level of each gene was given as the fold change relative to the transcriptional level of each corresponding gene in strain *A. baumannii* ATCC 19606 (assigned value of 1.0). All reactions were carried out in duplicate and experiments were repeated for three separate times.

### Statistical analysis

Gene expression differences between groups were analyzed using GraphPad Prism software V. 7.04 (GraphPad Software Inc., San Diego, CA), by one-way analysis of variance (ANOVA) followed by Dunnett's post hoc test correction for multiple comparisons and presented as mean ± standard deviations (SD). Comparison of categorical variables were assessed by Chi-square or Fisher’s exact tests using SPSS software for Windows version 22.0 (IBM Corp, Armonk, NY, USA). A *P *value < 0.05 was considered statistically significant.

### Nucleotide sequence accession numbers

Nucleotide sequences of the genes examined in this study are deposited in the NCBI database under the following GenBank accession numbers: *adeR*, MK344139-MK344153; *adeS,* MK355485-MK355499; *adeL*, MK318923-MK318937; *adeN*, MK344124-MK344138; *baeS*, MK344169-MK344183; *baeR*, MK344154-MK344168.

## Results

### Bacterial isolates, AST pattern and synergistic effect of PAβN

Of 95 isolates that were confirmed as *A. baumannii*, 76 (80%) of them were collected from intensive care unit (ICU), and the majority of samples were originated from respiratory secretions (Table 2).

**Table 2:** Demographics and clinical characteristics of patients involved in this study

Characteristics	No. (%)
Sex	
Male	60 (63%)
Female	35 (37%)
Age (year) (mean ± SD)	61.34 ± 16.1
Total length of hospitalization (days) (mean ± SD)	16.8 ± 12.05
Site of isolation	
Respiratory secretions	66 (69.4%)
Blood	12 (12.6%)
Wound	9 (9.5%)
Urine	3 (3.2%)
CSF	3 (3.2%)
Catheter	2 (2.1%)
Ward of isolation	
ICU	76 (80%)
Surgery	13 (13.7%)
Internal	4 (4.2%)
CCU	2 (2.1%)

Referring to AST results (Table 3); 89 (94%) isolates were considered as XDR, which all were resistant to carbapenems and among them 66 (74%) isolates were non-susceptible to tigecycline. The six remaining isolates were MDR. All isolates were revealed to be susceptible to colistin (≤ 1 µg/ml). In the phenotypic assessment of the efflux-based mechanism of resistance, all of the isolates were grown in the presence of PAβN (25 µg/ml). PAβN decreased the MIC of cefepime (256–128 to 64–32 µg/ml), gentamicin (256–96 to 64–24 µg/ml) and tigecycline (16–4 to 4–1 µg/ml) ≥ four-fold in 38 (40%) isolates, and also decreased the MIC of levofloxacin in only one isolate (four-fold) (32 to 8 µg/ml). All changes in the MIC and AST pattern of the aforementioned antimicrobials after the addition of PAβN are presented in Table 3. Among 38 isolates, some showed fold reduction in MIC of one antibiotic and some others showed fold reduction in MIC of two or three antibiotics. Among these antibiotics, PAβN had a higher effect on the MIC of tigecycline. However, the MIC of imipenem was not affected in the presence of PAβN (Table 4). Totally, 15 isolates including 13 isolates with > four-fold reduction in MIC of at least one of the over-mentioned antibiotics, and two isolates without any MIC reduction were selected for further sequence and expression analyses.

**Table 3:** Antimicrobial susceptibility profile of 95 isolates of *A. baumannii*, and MIC reductions of the antibiotics in exposure to PAβN

Antimicrobial agents	Disk diffusion			Without PAβN/with PAβN (25 µg/ml)					
	S no. (%)	I no. (%)	R no (%)	MIC range (µg/ml)	MIC_50_ (µg/ml)	MIC_90_ (µg/ml)	S no. (%)	I no. (%)	R no. (%)
Imipenem^a^	2 (2)	6 (6)	87 (92)	0.38–32/0.38–32	32/32	32/32	1 (1)/1 (1)	0/0	94 (99)/94 (99)
Meropenem	1 (1)	0	94 (99)	–	–	–	–	–	–
Doripenem	0	1 (1)	94 (99)	–	–	–	–	–	–
Cefotaxime	0	1 (1)	94 (99)	–	–	–	–	–	–
Ceftazidime	1 (1)	0	94 (99)	–	–	–	–	–	–
Cefepime^a^	1 (1)	7 (7)	87 (92)	3–256/3–256	256/**96**	256/256	1 (1)/1 (1)	0/0	94 (99)/94 (99)
Ampicillin-Sulbactam	12 (13)	22 (23)	61 (64)	–	–	–	–	–	–
Ticarcillin-clavulanic	1 (1)	0	94 (99)	–	–	–	–	–	–
Piperacillin-Tazobactam	0	0	95 (100)	–	–	–	–	–	–
Gentamicin^a^	5 (5)	16 (17)	74 (78)	0.75–256/**0.38**–256	96/**32**	256/256	4 (4)/4 (4)	6 (6)/**14 (15)**	85 (90)/**77 (81)**
Amikacin	11 (12)	7 (7)	77 (81)	–	–	–	–	–	–
Tobramycin	46 (48)	2 (2)	47 (50)	–	–	–	–	–	–
Tetracycline	6 (6)	22 (23)	67 (71)	–	–	–	–	–	–
Minocycline	76 (80)	12 (13)	7 (7)	–	–	–	–	–	–
Tigecycline^a^	–	–	–	0.016–16/0.016–**12**	4/**2**	12/**6**	25 (26)/**58 (61)**	31 (33)/**20 (21)**	39 (41)/**17 (18)**
Cotrimoxazole	1 (1)	0	94 (99)	–	–	–	–	–	–
Levofloxacin^a^	1 (1)	0	94 (99)	0.064–32/0.064–32	32/**12**	32/32	2 (2)/2 (2)	13 (14)/13 (14)	80 (84)/80 (84)
Ciprofloxacin	1 (1)	1 (1)	93 (98)	–	–	–	–	–	–
Gatifloxacin	2 (2)	0	93 (98)	–	–	–	–	–	–
Colistin^b^	–	–	–	0.047–1.0	0.75	1.0	–	–	–

Disks concentration: imipenem, 10 µg; meropenem, 10 µg; doripenem, 10 µg; cefotaxime, 30 µg; ceftazidime, 30 µg; cefepime, 30 µg; ampicillin-sulbactam, 10/10 µg; ticarcillin-clavulanic, 75/10 µg; piperacillin-tazobactam, 100/10 µg; gentamicin, 10 µg; amikacin, 30 µg; tobramycin, 10 µg; tetracycline, 30 µg; minocycline, 30 µg; trimethoprim/sulfamethoxazole, 1.25/23.75 µg; levofloxacin, 5 µg; ciprofloxacin, 5 µg; Gatifloxacin, 5 µg

PAβN, phenylalanine-arginine beta-naphthylamide; MIC, minimum inhibitory concentration; MIC_50_ and MIC_90_, MIC for 50% and 90% of the isolates, respectively. Changes in MIC and AST pattern after addition of PAβN, are shown in bold

^a^MIC of these antibiotics is determined with and without PAβN efflux pump inhibitor

^b^Since disk diffusion is not accepted for colistin AST, its MIC was assessed according to CLSI

**Table 4:** Thirty eight isolates with ≥ four-fold reduction in MIC of at least one antibiotic after addition of PAβN (25 µg/ml)

Fold reduction in MIC	Antibiotics (no.)				
	Imipenem^a^	Levofloxacin	Cefepime	Tigecycline	Gentamicin
4	–	1	11	18	7
5	–	–	3	3	2
6	–	–	3	2	1
7	–	–	–	–	1

Among 38 isolates, some showed fold reduction in MIC of one antibiotic and some others showed fold reduction in MIC of two or three antibiotics. Accordingly, the number of isolates for each fold reduction and each antibiotic is the total number

^a^No fold reduction was observed in MIC of imipenem after addition of PAβN

### Sequence analysis

Efflux pump genes were detected in all of the 95 isolates. Among regulatory genes, we could not amplify *adeS* using our designed primer pair. Sequence analysis of the regulatory genes revealed several single nucleotide polymorphisms (SNPs) in the *adeR*, *adeS*, *adeL*, *adeN*, *baeS* and *baeR* genes of the selected isolates. In order to compare the amino acid substitutions and polymorphisms, the reference strains *A. baumannii* AYE, ACICU, ATCC 19606 and ATCC 17978 were included in the analysis. AdeR had I120V and A136V, and AdeS had L172P, G186V, F214L, N268H, S280A, Q281D and Y303F polymorphisms as compared to the reference strains. AdeS component had the highest alteration in its amino acid sequence among all 15 isolates. AdeL had only Q262R mutation. All SNPs found in *adeN* were silent mutations with no changes in amino acid sequences. BaeS had S437T, S471N and P474S mutations, and BaeR revealed to have only S40N amino acid substitutions in some of the isolates. No insertion sequence (IS) was found. All amino acid substitutions are shown in Table 5.

**Table 5:** Expression of RND efflux systems, amino acid substitutions in regulatory genes and antimicrobial susceptibility pattern of 15 *A. baumannii* isolates with more than four-fold reduction in MIC of at least one antibiotic

Strain	Ward of isolation	Expression level			Amino acid substitutions						AST pattern	AST pattern without PAβN (µg/ml)/fold reduction in MIC with PAβN				
		*adeB*	*adeG*	*adeJ*	AdeR	AdeS	AdeL	AdeN	BaeS	BaeR		IMP	LVX	FEP	TGC	GEN
ATCC 19,606	–	1	1	1	–	–	–	–	–	–	–	–	–	–	–	–
M2	ICU	2.47	2.53	1.24	H158L	L172P,H189Y,R195G,V235I,S280A,Q281D,Y303F	Q262R	–	S471N,P474S	S40N	XDR	R(32)/–	R(32)/–	R(256)/6	R(8)/–	R(128)/4
M3	ICU	2.56	8.75	2.51	H158L	L172P,H189Y,R195G,V235I,S280A,Q281D,Y303F	Q262R	–	S471N,P474S	S40N	XDR	R(32)/–	R(12)/–	R(256)/6	R(12)/–	R(256)/7
M4	ICU	1.97	0.26	2.11	H158L	L172P,H189Y,R195G,V235I,S280A,Q281D,Y303F	Q262R	–	S471N,P474S	S40N	XDR	R(32)/–	R(32)/–	R(256)/6	R(8)/–	R(256)/5
M9	ICU	10.70	32.89	2.63	I120V,A136V	L172P,G186V,N268H,S280A,Q281D,Y303F	–	–	S437T	–	XDR	R(32)/–	R(32)/4	R(256)/4	R(8)/–	R(256)/6
M20	ICU	9.31	0.09	2.86	D93E, I120V,F132S	S46N, L172P, F214L,I257V,S280A,Q281D,Y303F,I331V	–	–	S471N	–	XDR	R(32)/–	I(4)/–	R(64)/–	R(8)/5	R(256)/–
M23	ICU	10.99	0.04	0.90	D93E, I120V,F132S	S46N, L172P, F214L,I257V, S280A,Q281D, Y303F	–	–	S471N	–	XDR	R(32)/–	I(4)/–	R(256)/4	R(12)/6	R(256)/–
M24	ICU	5.27	3.89	5.85	I120V,A136V	L172P,G186V,N268H,S280A,Q281D,Y303F,N342I	–	–	S437T	–	XDR	R(16)/–	R(8)/–	R(64)/–	R(16)/6	R(16)/–
M29	ICU	1.21	0.008	0.99	D93E, I120V,F132S	S46N, L172P, F214L,I257V, S280A,Q281D,Y303F,I331V	–	–	S471N	–	XDR	R(32)/–	R(12)/–	R(192)/–	R(12)/5	R(256)/–
M30	ICU	1.74	0.002	1.85	D93E, I120V,F132S	S46N, L172P, F214L,I257V, S280A,Q281D,Y303F,N342I	–	–	S471N	–	XDR	R(32)/–	I(4)/–	R(96)/–	R(8)/5	R(256)/–
M31	ICU	0.53	0.22	1.82	H158L	L172P,H189Y,R195G,S280A,Q281D,Y303F,V235I	Q262R	–	S471N,P474S	S40N	XDR	R(32)/–	R(16)/–	R(256)/5	R(16)/–	R(96)/–
M40	ICU	19.69	0.90	2.04	–	L172P,F214L, S280A,Q281D,Y303F	Q262R	–	S471N,P474S	S40N	XDR	R(32)/–	R(8)/–	R(64)/–	R(8)/–	R(256)/–
M42	ICU	16.44	42.51	2.02	–	L172P,F214L, S280A,Q281D,Y303F	Q262R	–	S471N,P474S	S40N	XDR	R(32)/–	R(8)/–	R(64)/–	R(8)/–	R(256)/–
M54	ICU	1.10	0.001	1.75	D93E, I120V,F132S	S46N, L172P, F214L,I257V, S280A,Q281D,Y303F,I331V, N342I	–	–	–	–	XDR	R(32)/–	I(4)/–	R(256)/5	S(2)/–	R(256)/–
M59	ICU	5.09	0.004	0.82	D93E, I120V,F132S	S46N, L172P, F214L,I257V, S280A,Q281D,Y303F,I331V, N342I	–	–	S471N	–	XDR	R(32)/–	I(4)/–	R(256)/5	R(8)/4	R(256)/–
M60	ICU	1.16	0.70	1.74	D93E, I120V,F132S	S46N, L172P, F214L,I257V, S280A,Q281D,Y303F,I331V, N342I	–	–	S471N	–	XDR	R(32)/–	R(32)/–	R(256)/–	I(4)/–	R(256)/5

AST, antimicrobial susceptibility testing; PAβN, phenylalanine-arginine beta-naphthylamide; MIC, minimum inhibitory concentration; IMP, imipenem; LVX, levofloxacin; FEP, cefepime; TGC, tigecycline; GEN, gentamicin

### Relative gene expression of RND efflux systems

**Fig. 1:**
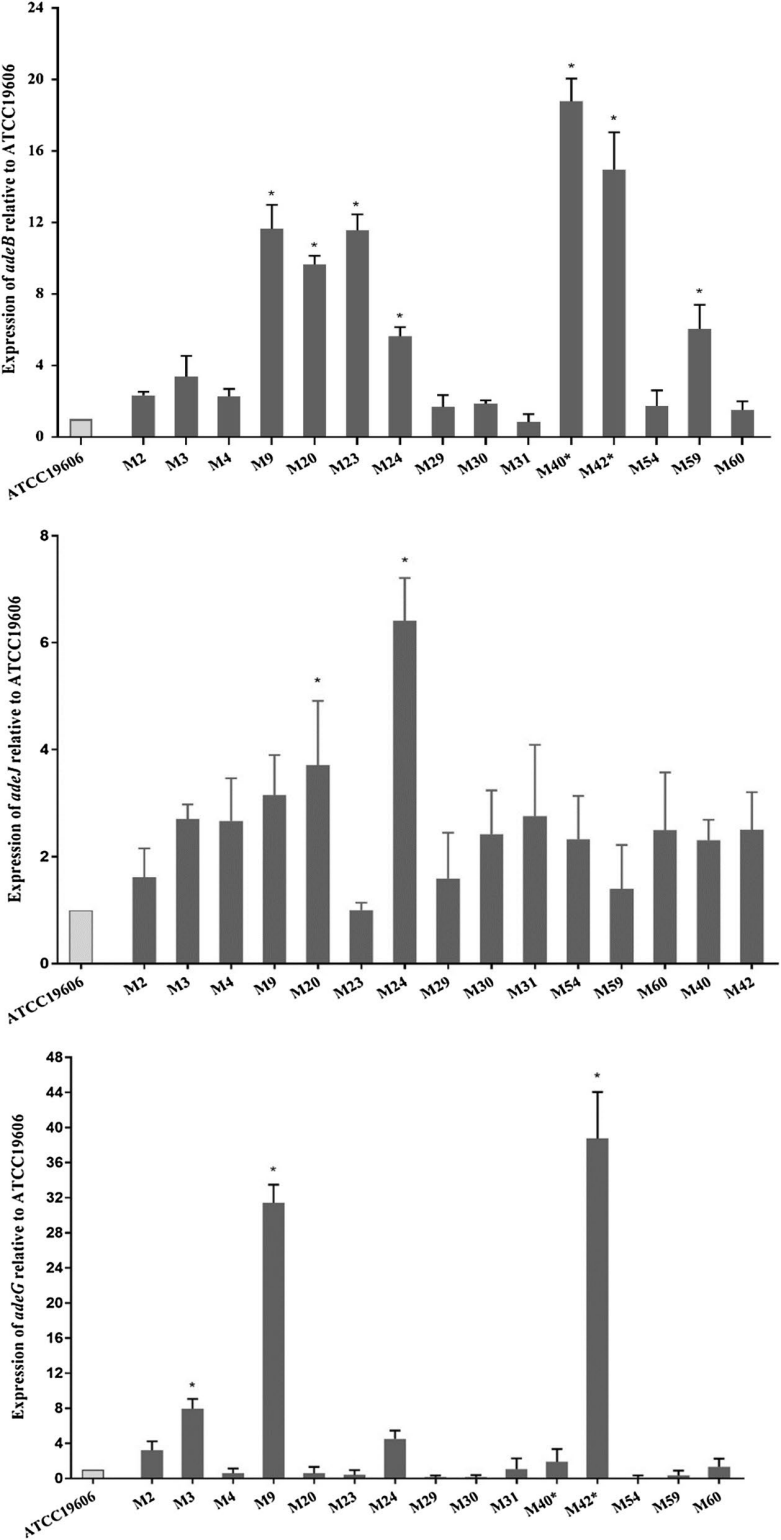
Relative expression of *adeB*, *adeG*, and *adeJ* genes by qRT-PCR. The relative expression level after being normalized to the expression of the reference gene *rpoB* were compared relative to that in the reference strain ATCC 19606. The bars represent the means and the error bars the standard errors for the average of the results from independent experiments. Statistical analysis was done by performing one-way ANOVA followed by Dunnett's post hoc test (*P *value < 0.05)

The RNA transcript of the major part of tripartite efflux systems (*adeB*, *adeG* and *adeJ*) in the selected isolates were determined by qRT-PCR in relative to that by ATCC 19606 reference strain. Seven, three and two isolates significantly (*P* < 0.05) overexpressed the *adeB* (5.09 to 19.69-fold)*, adeG* (8.75 to 42.51-fold) and *adeJ* (2.86 and 5.85-fold) genes, respectively (Fig. 1).

## Discussion

The impact of multiple resistance mechanisms in the success of *A. baumannii* as a notorious pathogen has led to confined treatment regimens for this tenacious microorganism [1]. In the present study, the majority of isolates showed an increased rate of resistance to all the first-line antibiotics in comparison to a previous study from Tehran, Iran [32]. Although carbapenems are the mainstay of treatment, all of our XDR isolates were resistant to carbapenems. Since carbapenem-resistant *A. baumannii* is listed as the “top priority” pathogen in the summit of critical resistant bacteria by WHO, its emergency for research and discovery to explore novel therapeutic options has been recently highlighted [33, 34].

Hitherto, there has been no consensus for the optimal treatment of *A. baumannii*-associated infections, in particular hospital-acquired pneumonia and bloodstream infections, caused by XDR isolates that are often carbapenem-resistant. Whereas treatment regimen should be made on the case-by-case basis of antimicrobial susceptibility; but considering the importance of early appropriate action, combination therapy is beneficial to target several resistance determinants. Despite dosage limitation due to the colistin nephrotoxicity, this agent is used as a backbone for salvage therapy of the carbapenem-resistant XDR-AB isolates. Colistin-based therapy in combination with sulbactam and tigecycline, in case of susceptibility, responds better than monotherapy. Fortunately, all of our isolates were susceptible to colistin. However, only 13% and 26% of the isolates were susceptible to ampicillin-sulbactam and tigecycline, respectively. The inconsistency in outcomes of colistin-based treatment have made it impotent. As an alternative, tigecycline as a drug of last resort for treatment of XDR-AB isolates confers a lower cure rate in cases with bloodstream infections due to low serum concentration, and high rates of non-susceptibility in the studies like ours [2, 33, 35, 36]. Consequently, approaching an evidence-based therapy is still controversial. Furthermore, it has been alarming that *A. baumannii* can develop drug resistance under the selective pressure both in vitro and through exposure with different antibiotics especially imipenem [5, 18, 37, 38]. Thus, with attention to concerns over rapidly growing number of resistant organisms, some strategies have been brought up to survey novel effective therapeutic trajectories to restore the efficacy of approved antibiotics. For instance, EPIs can hamper efflux activity and have the potential to be used in combination therapy. RND efflux systems have a major role in resistance to multiple categories of antibiotics, and has been verified via inactivation of efflux pump encoding genes [8–10]. Our results were in contrast to another study in which PAβN reduced the MIC of imipenem in 66% of imipenem-resistant *A. baumannii* isolates [39]. It is suggested that higher concentration of PAβN inhibitor can bypass efflux activity against imipenem. Considering that almost all of our isolates were resistant to imipenem with MIC of ≥ 16 µg/ml, resistance determinants such as reduced permeability of the outer membrane or production of carbapenemases could be involved in the carbapenem resistance in addition to efflux systems [33]. In our previous study, we detected OXA-type encoding genes in these isolates [40]. PAβN also affected the gentamicin-resistant phenotype of eight isolates. Moreover, this EPI reduced the MIC of cefepime- and levofloxacin-resistant isolates, but had no impact on their susceptibility patterns. Thus, the contribution of other resistance mechanisms can be deduced [33]. To be noted, PAβN remarkably restored tigecycline susceptibility (from 26 to 61%); accordingly, active multidrug efflux pumps conferred tigecycline non-susceptibility in our isolates but the type of the pump is not clarified yet [4]. Tigecycline non-susceptibility has been associated with three RND systems as already mentioned [2, 7, 36]. According to previous studies, AdeABC is the predominant pump conferring acquired resistance to a wide range of antibiotics. It is the only RND pump that extrudes aminoglycosides [8, 13, 14, 37, 41]. Although the role of this pump in carbapenem resistance is controversial, efflux activity was associated with reduced susceptibility to carbapenems under imipenem-selected stress [5]. AdeRS in the adjacent of AdeABC operon regulates it by transcribing in the opposite direction. Some putative mutations in AdeR are responsible for *adeB* overexpression: A91V and A136V in the signal receiver domain [3, 14], D20N in the phosphorylation site [5], and P116L at the first residue of the helix α5 [13]. Among these, A136V polymorphism in the signal receiver of AdeR regulator was detected in two *adeB* overexpressed isolates (M9 and M24) in our study. In AdeS, which is more prone to mutation, numerous point mutations can boost *adeB* expression: G30D located in the periplasmic loop [42], G103D alterations in the histidine kinase, adenylyl cyclase, methyl-accepting chemotaxis protein and phosphatase (HAMP) linker domain [14], G186V in the α-helix of the dimerization and histidine phosphotransfer (DHp) domain [3], and T153M in the histidine box [13]. In our study, two isolates (M9 and M24) with overexpression of *adeB* had G186V mutation, which can alter the conformation of AdeS DHp domain, and then stimulates overexpression of the AdeABC efflux pump [43]. Four of our isolates harbored H189Y located at the C-terminal of the DHp domain of AdeS, which can affect HK autokinase activity or RR phosphorylation [6]. Furthermore, the coexistence of A136V and G186V polymorphisms respectively in AdeR and AdeS components of two tigecycline-resistant isolates (M9 and M24) is noteworthy. M9 with a 10.70-fold increase in *adeB* expression level showed efflux pump activity for levofloxacin, cefepime and gentamicin antibiotics, and M24 with a 5.27-fold increase in *adeB* expression level showed efflux activity for tigecycline in the phenotypic assay. In line with our results, the coexistence of these two amino acid substitutions have been detected in both tigecycline-resistant and -susceptible isolates; hence, their detailed impact is a matter of controversy [3, 4, 6, 14]. The highest expression level of *adeB* was detected in M40 (19.69-fold) and M42 (16.44-fold) isolates; two XDR isolates without any reduction in MIC after PAβN addition. Additionally, these isolates had a few identical point mutations in each of AdeS, BaeR and BaeS regulators, and had no mutations in AdeR. They harbored none of the renowned mutations and all the detected substitutions were also found in isolates with no pump overexpression. Furthermore, disrupted *adeS* by ISA*ba1*can lead to tigecycline non-susceptibility and even other antibiotics by enhancing the AdeABC overexpression [6, 11, 12, 41]. However, this insertion was not detected in our studied isolates.

Regarding the strict regulation of resistance mechanisms under external pressures, the transcriptional regulators were introduced as promising drug targets to overcome resistance. However, the results obtained by Trebosc et al. revealed that there are AdeR-unrelated mechanisms mediating tigecycline resistance, which made AdeR an insufficient target for adjuvant therapy [17]. Tigecycline non-susceptibility can occur as a result of synergistic contribution of AdeIJK with AdeABC [10], while AdeABC has superior influence [18, 38]. The AdeIJK efflux pump is species-specific and contributes to intrinsic resistance to various antibiotics [10]. It is tightly regulated by the product of *adeN* gene in ca. 800 kb away from the AdeIJK operon transcribing in the same direction [16, 44]. Since high-level expression of this pump is toxic for *A. baumannii*, AdeN represses AdeIJK and its disruption diminishes susceptibility following a tolerable expression level. A premature stop codon in the helix α9 sequence at position 211 within the dimerization domain inactivates AdeN [16]. In another study, three types of insertions including ISA*ba1* leading to *adeN* inactivation were detected [11]. Overall, the expression level of *adeJ* was very slight in our isolates confirming the theory of its lethality for the host. M20 and M24 isolates with minor increases in the expression of *adeJ* were tigecycline-resistant with pump activity (Table 5). Therefore, the role of other mechanisms in regulation of the AdeIJK operon cannot be ruled out.

The BaeSR is a global regulator and has been associated with tigecycline resistance by controlling AdeIJK and AdeABC pumps. It has been reported that the function of BaeSR occurs through a cross-talk with AdeRS, suggesting the overlap of these two TCS regulons [17, 19–21]. Accordingly, we assessed the BaeSR sequence for any mutation that might be effective in efflux pump expression. In two isolates (M9 and M24) with increased expression level in *adeB* and *adeJ*, we found N268H and S437T polymorphisms in AdeS and BaeS, respectively. These two polymorphisms were not found in the isolates with no increase in the expression of efflux pumps. So far as we know, this is the first investigation on BaeSR and its probable role in the resistance of *A. baumannii* isolates in Iran. A better understanding of the dynamic interaction between AdeRS and BaeSR in the regulation of RND efflux pumps of *A. baumannii* merits further investigations.

The contribution of AdeFGH to acquired resistance has been proved second to AdeABC. Its overexpression confers decreased susceptibility to several agents. The *adeL* regulates AdeFGH operon in the upstream of it transcribing in the opposite direction. Deletion of the 11 C-terminal residues, T319K and V139G in the signal recognition domain confer increased expression of *adeG* [9]. Only Q262R amino acid substitution was found in our isolates. Isolate M42 with the highest expression level (42.51-fold) displayed Q262R substitution in AdeL; but isolate M9 with a 32.89-fold increase in *adeG* expression showed no alteration. Thus, involvement of other mechanisms is proposed.

The resultant findings of our study elucidated that tigecycline is a substrate for the three aforementioned RND pumps; however, some tigecycline-resistant isolates revealed no pump overexpression. Additional mechanisms contribute to the tigecycline resistance, as previously described [11, 17]. Among the studied isolates, only isolate M54 was tigecycline-susceptible with no efflux activity and no increase in efflux pump expression, but surprisingly showed a five-fold reduction in the MIC of cefepime indicating efflux activity. Furthermore, two tigecycline-resistant isolates (M29 and M30) with efflux activity for tigecycline and no overexpression of the RND efflux pumps revealed the possibility that other efflux systems play a role in resistance to tigecycline [4].

## Conclusions

To our knowledge, this is the first comprehensive study so far; investigating the gene expression of three prominent RND efflux pumps and the genetic mutations in their regulators among XDR clinical isolates of *A. baumannii* in Iran. Our results revealed that regardless of the contribution of RND-type efflux pumps to resistance against multiple classes of antibiotics especially tigecycline, the increased MIC of antibiotics was not constant as a result of the pump overexpression. Overexpression of efflux pumps supports *A. baumannii* to cope with external stresses and survive in the harsh hospital environments. Evaluation of efflux activity using non-specific EPIs is difficult because of the multiplicity of the pumps. Moreover, the evolution of hypermutator phenotypes and complex sets of resistant determinants have been made it labyrinthine to gain insights into regulatory mechanisms. Consequently, eradication of the newly emerged XDR-AB isolates seems to be strain-dependent. In conclusion, the precise interpretation of discrepant findings obtained with efflux pumps’ regulation and expression require (1) characterization of every single mutation in cognate regulators of RND efflux systems, (2) constructing point mutant or its deleted counterpart for pairwise comparison with the parental strain, and (3) development of clinically useful EPIs with an efficient dosage for circumventing efflux pumps. These strategies will be the subject of our future researches, as exerting severe infection control measures and novel therapeutic options to overcome nosocomial XDR-AB strains are desperately needed.

## Data Availability

All data generated or included during this study are featured in this published article.

## Abbreviations

XDR-AB: Extensively drug-resistant *Acinetobacter baumannii*
RND: Resistance-nodulation-cell division
Ade: *Acinetobacter* Drug efflux
MDR: Multidrug resistant
TCS: Two-component system
SK: Sensor kinase
RR: Response regulator
AST: Antimicrobial susceptibility testing
MIC: Minimum inhibitory concentration
CLSI: Clinical and laboratory standards institute
EPI: Efflux pump inhibitor
PAβN: Phenylalanine-arginine beta-naphthylamide
PCR: Polymerase chain reaction
qRT-PCR: Quantitative real-time PCR
NCBI: National center for biotechnology information
BLAST: Basic local alignment search tool
CCU: Coronary care unit
ICU: Intensive care unit
CSF: Cerebrospinal fluid
IS: Insertion sequence
IMP: Imipenem
LVX: Levofloxacin
FEP: Cefepime
TGC: Tigecycline
GEN: Gentamicin
